# Validation of the Orgasm Rating Scale in Context of Sexual Relationships of Gay and Lesbian Adults

**DOI:** 10.3390/ijerph19020887

**Published:** 2022-01-13

**Authors:** Pablo Mangas, Reina Granados, Oscar Cervilla, Juan Carlos Sierra

**Affiliations:** 1 Mind, Brain and Behaviour Research Centre (CIMCYC), University of Granada, 18011 Granada, Spain; pablomangas@ugr.es (P.M.); ocervilla@ugr.es (O.C.); jcsierra@ugr.es (J.C.S.); 2 Department of Nursey, Health Sciences Faculty, University of Granada, 18071 Granada, Spain

**Keywords:** Orgasm Rating Scale, subjective orgasm experience, gay men, lesbians, reliability, validity evidence

## Abstract

Currently, no validated instrument exists for assessing the subjective experience of orgasm in the gay population. The Orgasm Rating Scale (ORS), previously validated in the heterosexual population, comprises four dimensions: Affective, Sensory, Intimacy, and Rewards. This study validated it for sexual relationships in the gay population by obtaining its factorial invariance by sexual orientation and sex, its internal consistency reliability, and evidence of validity in its relationship with other variables. We assessed 1600 cisgender Spanish adults–heterosexuals, gays, and lesbians–divided into 4, sex-based groups of 400 each, according to the Kinsey scale scores. Participants reported recent experiences of orgasm in the context of sexual relationships and responded to the ORS and other scales assessing attitude toward sexual fantasies and sexual functioning. The ORS structure showed a strict multigroup-level invariance by sexual orientation and sex, confirming its four-dimensional structure. The subjective orgasm intensity was associated with a positive attitude toward sexual fantasies and sexual functioning. Scores obtained on the Affective, Intimacy, and Rewards dimensions confirmed the ability to discriminate between gay people with and without orgasmic difficulties. The ORS’s Spanish version presents good psychometric properties as a validated scale to evaluate the subjective experience of orgasm in the gay population.

## 1. Introduction

From a biopsychological perspective, orgasm is described as the moment of maximum sexual pleasure, in which rhythmic contractions of the perineal organs occur, accompanied by cardiovascular and respiratory changes, as well as release of sexual tension [1]. This sensation of pleasure creates an altered state of consciousness, resulting in a sense of well-being and satisfaction [2]. One dimension of orgasm that has not been accorded adequate attention is its subjective experience, which refers to the perception, appraisal, and/or sensation at the psychological level [3,4].

The relevance of the subjective experience of orgasm in the context of sexual relationships with a partner lies in its association with sexual satisfaction [5,6], as well as being related to other indicators of sexual health, such as erotophilia, sexual desire, or sexual arousal [3,7,8]. In this context, it has been observed that people who report difficulties related to orgasm experience it with less intensity at a subjective level [5].

The sexuality of gay people has been studied less than that of heterosexuals, and, sometimes, it has been left out of investigations [9]. However, the World Association for Sexual Health [10] defends the right to have pleasurable and satisfying sexual experiences regardless of a person’s sexual orientation. Similarly, the Yogyakarta Principles [11] (p. 22) establish in Article 17, “the right to the highest attainable standard of health without discrimination on the basis of sexual orientation or gender identity”, where ensuring the sexual and reproductive health of sexual minorities assumes a special role. This justifies the need for research on sexual minorities.

Numerous studies have compared dimensions of sexuality between gay and heterosexual populations, but in many cases, the results are not supported by invariant measures of sexual orientation. Some examples, where comparisons have been made between groups with different sexual orientations—without the support of invariant measures –refer to sexual functioning [12], dyadic and solitary sexual desire [13], involvement in sexual-risk behaviors [14], attitudes associated with gender roles [15], perceptions of physical attractiveness and body satisfaction [16], stereotypes about masculinity/femininity [17], motivations toward fatherhood [18], attitudes toward homosexuality [19], or sexual fantasies [20]. That is, although it is common to compare different dimensions of sexuality between people with different sexual orientations, hardly any studies have focused on the invariance of the measures used for such comparisons.

Moreover, few studies have examined the subjective experience of orgasm in sexual minorities. Basically, their frequency has been addressed, observing an orgasmic gap in the male–female binary pole to the detriment of women [21,22,23]. Although there are no differences in orgasmic frequency for men of different sexual orientations [24], there are differences for women: lesbians are significantly more likely to reach orgasm than bisexuals and heterosexuals [25,26].

An instrument that allows for an assessment of the subjective experience of orgasm is the Orgasm Rating Scale (ORS) [27,28], which has been validated in the Spanish population in the context of heterosexual relationships by Arcos-Romero et al. [5]. Based on the Multidimensional Model of the Subjective Experience of Orgasm proposed by Mah and Binik [4], the Spanish version of the ORS proposes the multidimensionality of the subjective experience of orgasm: (1) Affective or feelings experienced; (2) Sensory or physiological sensations; (3) Intimacy or closeness; (4) Rewards or consequences derived from orgasm [5,29]. The ORS has shown good psychometric properties in the context of heterosexual relationships, with a range of internal consistency between 0.78 (Intimacy) and 0.93 (Sensory). Regarding validity evidence, its scores have been significantly and positively related to sexual satisfaction and erotophilia. Likewise, it can differentiate between people with and without orgasmic difficulties [5]. Additionally, this version was found to be invariant by sex and educational level in the heterosexual population [29].

Therefore, the present study aims: (1) to examine the factorial invariance by sexual orientation and sex of the Spanish version of the ORS proposed by Arcos-Romero et al. [5], in order to be able to compare the subjective experience of orgasm in the realm of sexual relationships between gay and heterosexual men and women; (2) to estimate its internal consistency reliability; (3) to obtain evidence of the validity of its measures based on the relationship with other constructs (i.e., positive attitude toward sexual fantasies and sexual functioning); (4) to estimate its ability to discriminate between gay people with and without orgasmic difficulties.

In this regard, it is expected: (1) that the ORS is invariant by sexual orientation and sex [29]; (2) that, in the gay population, its scores positively correlate with positive attitudes toward sexual fantasies and sexual functioning [5]; (3) that gay people compared to heterosexuals [30], and lesbians compared to gay men [6,29], subjectively experience orgasm with greater intensity; (4) finally, that gay men and women with orgasmic difficulties obtain lower scores on the ORS than those without difficulties [5].

## 2. Materials and Methods

### 2.1. Participants

By means of a non-probabilistic incidental sampling, 1600 Spanish adults were evaluated, divided into 2 subsamples: (1) gay people (*n* = 800; 400 men and 400 women), and (2) heterosexual people (*n* = 800; 400 men and 400 women), with an age range between 18 and 63 years (*M* = 32.11; *SD* = 10.24). The following inclusion criteria were considered: (a) Spanish nationality; (b) being at least 18 years old; (c) being cisgender; (d) having had orgasm experiences in the last 6 months in the context of sexual relationships (with same-sex partners, gay participants; and with different-sex partners, heterosexual participants). The sociodemographic characteristics of the participants are organized by sexual orientation and sex in Table 1.

### 2.2. Instruments

Sociodemographic and Sexual History Questionnaire, in which information was requested on sex, age, nationality, educational level, age of first sexual relationship (oral, vaginal, or anal), partner relationships, partner’s duration, sexual activity, number of sexual partners, and method employed to obtain the last orgasm in sexual relationships with another person.

The Kinsey Scale [31], which assesses sexual orientation from exclusively heterosexual to exclusively homosexual. Its items made it possible to group the subjects evaluated into the 2 subgroups: (1) gays (subjects who selected the *exclusively homosexual* option), and (2) heterosexuals (subjects who selected the *exclusively heterosexual* option).

The Spanish version of the ORS [28] by Arcos-Romero et al. [5], which assesses the subjective experience of orgasm in the context of sexual relationships in the presence of a partner by means of 25 items distributed among four factors: Affective (e.g., “Elated”), Sensory (e.g., “Flooding”), Intimacy (e.g., “Loving”), and Rewards (e.g., “Peaceful”). It uses a 6-point Likert-type scale to quantify how well each of the 25 adjectives describe their most recently experienced orgasm, where 0 means *does not describe it at all* and 5 means *describes it perfectly* so that the higher the score, the greater the subjective orgasm intensity.

The Spanish version of the Hurlbert Index of Sexual Fantasy (HISF) [32] by Sierra et al. [33], which consists of 10 items (e.g., “I think sexual fantasies are healthy”) that are answered on a 5-point Likert-type scale from 0 (*never*) to 4 (*all of the time*). High scores indicate a positive attitude toward sexual fantasies. It has been shown to be invariant by sex, age, and educational level, and its internal consistency reliability is 0.94. Additionally, it can differentiate between individuals with and without difficulties in sexual functioning, as well as expected correlations with related constructs [33]. In the present study, the ordinal alpha coefficient of the scale was 0.89, both for the sample of gay and heterosexual individuals.

The Spanish version of the Arizona Sexual Experience Scale (ASEX) [34] by Sánchez-Fuentes et al. [35], whose five items assess sexual functioning during the past seven days associated with drive, arousal, erection (in men), vaginal lubrication (in women), orgasm, and satisfaction from orgasm. They are answered on a 6-point Likert scale (from 1 or *good functioning* to 6 or *poor functioning*). Their scores were inverted, so that higher scores indicate better sexual functioning. The obtained unifactorial solution shows good internal consistency reliability in men (α = 0.81) and in women (α = 0.79). Sánchez-Fuentes et al. [35] reported evidence of convergent validity by significantly correlating their scores, in the expected sense, with other measures of sexual functioning, erotophilia, propensity to become sexually aroused/inhibited, and objective and subjective sexual arousal. In the present study, the ordinal alpha coefficient was 0.72 in the heterosexual male sample, 0.76 in the gay male sample, 0.80 in the heterosexual female sample, and 0.85 in the lesbian sample.

### 2.3. Procedure

Participation was voluntary and anonymous. Online questionnaires were used, which is a procedure equally valid as the traditional paper-and-pencil procedure in studies of this type [36,37]. Using the Open-Source LimeSurvey platform, located on the servers of the University of Granada, an electronic version of the survey was disseminated through Facebook^®^. Payment was made to the virtual platform to promote the survey from 5 October 2020 to 11 December 2020, among adults throughout Spain.

Participants were informed of the purpose and voluntary nature of the study, the characteristics of the evaluation, and what their participation implied. They were assured of anonymity, data protection, and the confidentiality of responses, that is, the use of data for the sole purpose of research and scientific dissemination. The anonymity of the participants was ensured from the beginning to the end of the data collection, so that the identity of each participant was protected at all times. At no time was personal information required by which a participant could be identified (i.e., name, surname, ID card or passport). They expressed their agreement to participate in the research via an informed consent form, which also indicated the purpose of the study.

Different procedures were used to control online samples to prevent duplicate, fraudulent, or bot-generated responses, including tracking the IP address of each of the accesses to the questionnaire battery (being impossible to identify someone by IP, so confidentiality was guaranteed), a numerical CAPTCHA in the form of a randomized arithmetic calculation at the beginning of the questionnaire, as well as tracking URLs posted in suspicious locations [38,39]. The data were thoroughly reviewed to negate cases with inconclusive responses or abnormal patterns. The approximate time to complete the questionnaire battery was 20 min. The study was approved by the Human Research Ethics Committee of the University of Granada.

### 2.4. Data Analysis

Missing data were imputed through an algorithm for non-parametric distributions by creating a random forest model for each variable. To determine whether the factor structure proposed by Arcos-Romero et al. [5] for the ORS constitutes an invariant measure in gay and heterosexual men and women, a successive multi-group Factorial Invariance by sexual orientation and sex was conducted, defining four groups: gay men, lesbian women, heterosexual men, and heterosexual women. The progressive invariance (configural, weak, strong, and strict) of the four-factor model was tested. We used the Weighted Least Squares Mean Adjusted (WLSM) estimation method, following recommendations [40]. The WLSM is a robust estimator of non-compliance with multivariate normality and designed for ordinal/categorical data [41].

In relation to the fit indicators, the following were considered: the Root Mean Square Error of Approximation and its 90% confidence interval (RMSEA) [42], the Comparative Fit Index (CFI) [43], and the Tucker-Lewis Index (TLI). To evaluate the fit of the CFA, the following criteria were considered: RMSEA < 0.08, CFI and TLI > 0.90 [44,45], and the difference between the values of the comparative index in the CFI [43], considering that, if the difference between 2 nested models has a value in the CFI greater than 0.01 in favor of the model with fewer restrictions, the model with more restrictions would be rejected [46]. Internal consistency reliability was estimated using the ordinal alpha coefficient [47].

Using the multivariate analysis of covariance (MANCOVA), ORS scores were compared by sex in the gay sample, as well as between gay and heterosexual men, and between lesbians and heterosexual women. Following this, Pearson’s correlations were used to relate the ORS dimension scores to positive attitudes toward sexual fantasies and sexual functioning. Finally, Bayesian analyses were conducted to examine the ability of the ORS to discriminate between gay men and women with and without orgasmic difficulties. A total of 2 -sex and age- matched groups were formed: 56 cases without orgasmic difficulties (*M*_age_ = 31.70; *SD* = 10.09) and 56 cases with orgasmic difficulties (*M*_age_ = 31.71; *SD* = 10.12). Subjects were considered to have difficulties if they indicated a score of 5 (*very difficult* and *very unsatisfying*, respectively) or 6 (*never reach orgasm* and *can’t reach orgasm*, respectively) on items 5 and 6 of the ASEX. In addition to these statistical considerations, Fisher’s Bayesian ANOVA analysis was applied to examine any differences as recommended by Ruíz-Ruano and López-Puga [48,49], and log-rank was employed for ease of interpretation. An *r*^JZS^ = 0.71 was used. According to Jeffreys [50], a more robust result would be away from zero if the following intervals are contemplated: 1–3 anecdotal, 3–10 substantial, 10–30 strong, 30–100 very strong, and >100 decisive.

The R^®^ environment (version 3.6.3) (The R Fundation, Vienna, Austria) [51] was used to perform the analyses using its RStudio^®^ interface (version 1.2.5042, RStudio PBC, Boston, MA, USA) [52]. The following freely R packages were used: missForest (version 1.4) [53] for missing data imputation, available from the CRAN repository (https://cran.r-project.org/ accessed on 6 May 2021); Psych (version 1.9.12.31) [54] for calculating ordinal alpha, available from the CRAN repository (https://cran.r-project.org/, accessed on 6 May 2021); lavaan for invariance [55], available from the CRAN repository (https://cran.r-project.org/, accessed on 6 May 2021); and tidyBF (version 0.4.0) [56] available from the CRAN repository (https://cran.r-project.org/, accessed on 6 May 2021), for Bayesian analyses.

## 3. Results

### 3.1. Measurement Invariance of the ORS across Sex and Sexual Orientation

Measurement invariance by sex and sexual orientation was tested for the four-factor model [5] and the results are shown in Table 2. The ΔCFI between the constrained and unconstrained models was below 0.01, indicating that strict invariance was supported according to Cheung and Rensvold [46]. The results support configural, weak, strong, and strict invariance of the four-factor model across sex and sexual orientation.

### 3.2. Reliability

To examine the reliability of the four subscales of the ORS in the four groups (gay and heterosexual men and women), we calculated ordinal’s alpha coefficients. The coefficients ranged from 0.82 to 0.94 (Table 3).

### 3.3. Differences across Sex and Sexual Orientation

The MANCOVA was used to compare the 4 subscales of the ORS between (1) gay men and lesbians, (2) gay men and heterosexual men, and (3) lesbians and heterosexual women, controlling for age, educational level, current partner or not, age of first sexual relationship (oral, vaginal, or anal), number of lifetime sexual partners, and the method used to reach the last orgasm in sexual relationships with another person.

As for gay people, having a partner (Wilk’s lambda = 0.83; *F*(4, 768) = 40.44, *p* < 0.001; *ƞ*^2^ = 0.17), the number of sexual partners (Wilk’s lambda = 0.97; *F*(4, 768) = 6.25, *p* < 0.001; *ƞ*^2^ = 0.03), and the method used to reach the last orgasm in sexual relationships with another person (Wilk’s lambda = 0.96; *F*(4, 768) = 7.28, *p* < 0.001; *ƞ*^2^ = 0.04) were significant multivariate covariates. Sex had a main effect on the subjective experience of orgasm (Wilk’s lambda = 0.93; *F*(4, 768) = 14.51, *p* < 0.001; *ƞ*^2^ = 0.07). The inter-subject effect on the subjective experience of orgasm is shown in Table 4. Significant differences are observed in all of the dimensions of the ORS in favor of women, except for Rewards.

However, in the male sample, age (Wilk’s lambda = 0.99; *F*(4, 767) = 2.59, *p* = 0.036; *ƞ*^2^ = 0.01), having a partner (Wilk’s lambda = 0.86; *F*(4, 767) = 32.3, *p* < 0.001; *ƞ*^2^ = 0.14), the number of sexual partners (Wilk’s lambda = 0.97; *F*(4, 767) = 6.4, *p* < 0.001; *ƞ*^2^ = 0.03), and the method used to reach the last orgasm in sexual relationships with another person (Wilk’s lambda = 0.96; *F*(4, 767) = 8.28, *p* < 0.001; *ƞ*^2^ = 0.04) were significant multivariate covariates. Sexual orientation had a main effect on the subjective experience of orgasm (Wilk’s lambda = 0.98; *F*(4, 767) = 3.71, *p* = 0.005; *ƞ*^2^ = 0.02). Finally, in the female sample, educational level (Wilk’s lambda = 0.99; *F*(4, 773) = 2.41, *p* = 0.048; *ƞ*^2^ = 0.01) and having a partner (Wilk’s lambda = 0.89; *F*(4, 773) = 23.89, *p* < 0.001; *ƞ*^2^ = 0.11) were significant multivariate covariates. Sexual orientation had a main effect on the subjective experience of orgasm (Wilk’s lambda = 0.97; *F*(4, 773) = 6.87, *p* < 0.001; *ƞ*^2^ = 0.03). The inter-subject effect on the subjective experience of orgasm is shown in Table 5. Significant differences were observed, according to sexual orientation, in the Rewards dimension in the case of men, in favor of heterosexuals, and in the Intimacy dimension in the case of women, in favor of lesbians.

### 3.4. Sources of Validity Evidence Based on the Relations with Other Variables

As shown in Table 6, in general, the bivariate correlations, although low, were statistically significant in the expected direction, except in the case of men, especially in the Intimacy subscale.

**Figure 1:**
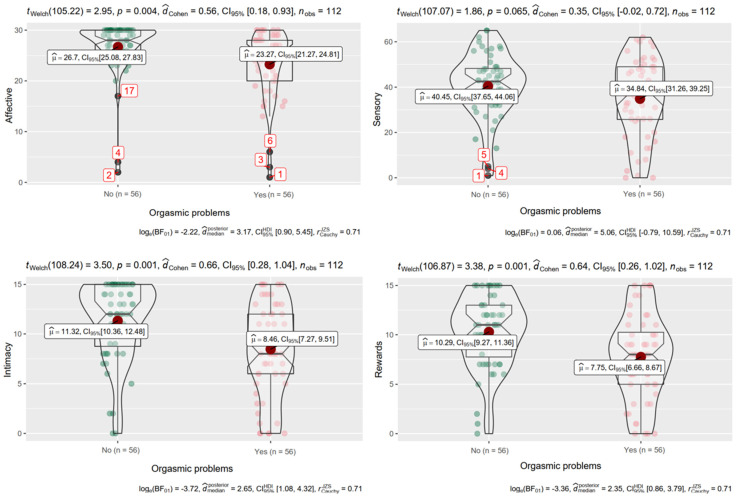
Distribution of data, for each of the four dimensions of the ORS, in the group with and without orgasmic problems. Note. Red dot indicates the population mean (µ) of that group. Bayesian results for the differences are shown below each figure.

Finally, evidence of discriminant validity was provided by examining the ability of the ORS to differentiate between gay people with and without orgasmic difficulties. As shown in Figure 1, both groups differed significantly in the Affective [*t_Welch_* (105.22) = 2.95, *p* = 0.004, *d_Cohen_* = 0.56, log_e_(BF_01_) = −2.22], Intimacy [*t_Welch_*(108.24) = 3.50, *p* = 0.001, *d_Cohen_* = 0.66, log_e_(BF_01_) = −3.72], and Rewards dimensions [*t_Welch_* (106.87) = 3.38, *p* = 0.001, *d_Cohen_* = 0.64, log_e_ (BF_01_) = −3.36], showing higher scores in the subjective experience of orgasm in subjects without orgasmic difficulties. In the Sensory dimension, no significant differences were obtained (*p* = 0.065), although a clear tendency toward the hypothesized differences can be observed.

## 4. Discussion

This study aimed to validate the Spanish version of the ORS in the context of homosexual relationships, previously validated to assess the subjective experience of orgasm in the context of heterosexual relationships by Arcos-Romero et al. [5]. The scale presented adequate psychometric properties in the gay population, also allowing for a bias-free comparison between gay and heterosexual people. The results obtained represent an advancement in the comparisons between both populations, as traditionally the measures used to evaluate different constructs in sexuality are invariant between the gay and heterosexual populations are not justified. Therefore, it is relevant to demonstrate the invariance of the instruments used [57].

The structure of the ORS, validated by Arcos-Romero et al. [5] in the context of heterosexual relationships, was found to be invariant between heterosexual and gay men and women. Thus, the four factors that constitute the scale (Affective, Sensory, Intimacy, and Rewards) enable us to characterize orgasm in homosexual relationships in a multidimensional way. Its measures reached strict levels of invariance by sex, as had already been established by Arcos-Romero and Sierra [29] in heterosexual people, and sexual orientation. The invariance of the ORS structure is relevant from a clinical perspective, as it allows for the comparison of the subjective experience of orgasm between partners, not only when the couple is heterosexual, but also when it is composed of members of the same sex. This is essential for ascertaining what facilitates a healthy sexuality in the non-heterosexual group, which has been traditionally ignored in related studies.

In reference to the reliability of the ORS, its subscales obtained adequate ordinal alpha values, in line with those previously reported in heterosexual population by Arcos-Romero et al. [5]. In the present study, in both gay and heterosexual men and women, reliability was slightly lower for the Intimacy and Rewards subscales compared to Affective and Sensory. The coefficients ranged from 0.82 (Intimacy in heterosexual women) to 0.94 (Sensory in lesbians), as in the heterosexual population, where the reliability reported by Arcos-Romero et al. [5] and Arcos-Romero and Sierra [29] follows a similar pattern.

Once the invariance by sex and sexual orientation of the ORS measures was obtained, the scores of its four dimensions were compared between both sexes in the gay sample, and between gays and heterosexuals. In gay individuals, in relation to sex–after controlling for the effect of having a partner, the number of sexual partners, and the method used to reach the last orgasm in sexual relationships with another person–significant differences were observed in all dimensions of the ORS (except for the subscale Rewards), so that lesbians experience orgasm with their partners more intensely than gay men. These results are similar to those of Arcos-Romero and Sierra [6,29] in the heterosexual population, as they report that heterosexual women, compared to men, show greater intensity in the subjective experience of orgasm. Similarly, in the study by Sierra et al. [58], the assessment of the subjective experience of orgasm resulting from masturbation was higher in women, both lesbians and heterosexuals. These sex differences in the intensity of the subjective experience of orgasm could be explained by the fact that women have a larger repertoire for describing their orgasmic sensations or rate more specific aspects of orgasm compared to men [6]. Another possible explanation refers to women, as compared to men, having a greater perception of the experience of orgasm in different parts of the body.

Regarding sexual orientation, after controlling for the effects of age, having a partner, the number of sexual partners, and the method used to reach the last orgasm in sexual relationships with another person, heterosexual men, compared to gay men, showed higher scores in the Rewards dimension of the subjective experience of orgasm. This indicates that they attach greater importance to the effects or consequences of orgasm obtained in the context of a couple relationship. This may imply that heterosexual men instrumentalize their sexual relationships to a greater extent on the basis of the consequences of the same (e.g., to relax or calm down), distinct from gay men, who do not attach so much importance to the consequences, focusing on the process or course of the sexual relationship, and not so much on the result of the same. As for women, once the effects of the level of education and having a partner were controlled for, it was observed that lesbians scored higher on the Intimacy dimension. Women with same-sex partners are characterized by high levels of emotional closeness compared to heterosexual couples [59], as well as by a high degree of intimacy [60], which may lead them to value the Affective, Sensory, and Intimate aspects to a greater extent than heterosexual couples. These results are consistent with studies indicating that in male couples, difficulties associated with intimacy may be caused by implicit and restrictive male gender norms [61,62], emotional disconnection [63], or feelings of competition between men [64]. Lesbians, generally, display higher levels of communication about their sex lives [65], which strengthens intimacy between them and provides them with a better dyadic adjustment [30,66].

In the comparisons discussed above, some variables were controlled for their potential effects on the subjective experience of orgasm. While the variable having a partner was significant in all of the comparisons–both by sex and sexual orientation–age, level of education, number of sexual partners, and method used to obtain the last orgasm in sexual relationships with another person were only significant in some of the comparisons. Evidence is available for the direct association between different aspects of partner relationships and the experience of orgasm, such as love [67], intimacy [68], or the duration of the relationship itself [69]. It is known that interpersonal variables in the context of the couple play a relevant role in explaining the subjective experience of orgasm, in both men and women [6]. Thus, the intensity of the subjective experience of orgasm declines with age [29], more markedly in men, in whom sexual functioning worsens more noticeably [70,71]. Educational level has a significant effect on the subjective experience of female orgasm. In relation to this, it has been reported that women with a low educational level have higher rates of sexual problems [72,73,74], specifically, poor orgasm experience [75]. This may be because low educational attainment is often associated with high levels of inappropriate sexual beliefs [76] and lower accessibility to quality sexual education, as most of these educational experiences occur in a formal academic context [77], which are essential to prevent infections and related difficulties [78]. The number of sexual partners also had a significant effect on the subjective experience of orgasm in comparisons by sex in the gay group and by sexual orientation, in men. Generally, it is men who tend to report a higher number of sexual partners, both in gay [30,66] and heterosexual [66,79] populations—a fact that could respond to the phenomenon of sexual double standard (see Álvarez-Muelas et al. [36,80] and Endendijk et al. [81]). Finally, the method used to obtain the last orgasm in sexual relationships with another person turned out to be a significant variable in the comparison by sex within the group of gay people, and by sexual orientation, in men. In this sense, it has been observed that the highest percentage of heterosexual men reach orgasm through vaginal penetration [82], while in gay men, although penetration also predominates, orgasm is reached through other alternative ways [83].

As for the validity evidence based on the relationships of ORS scores with other related psychosexual variables, generally, the correlations were statistically significant. Some of them showed similarities in men and women. Thus, sexual arousal was significantly associated with the four ORS dimensions, in a similar way in gays and lesbians. Something similar happened with satisfaction with orgasm; its correlations were consistent in both sexes. Although with small differential nuances between men and women, the other components of the sexual response (desire, erection/lubrication, and orgasm) were also associated with the subjective experience of orgasm, which shows that this psychological dimension of orgasm must be understood and explained together with the components of the sexual response, as already evidenced by Arcos-Romero and Sierra [6].

It is noteworthy that, while vaginal lubrication correlates with all dimensions of the ORS, erection only correlates with the Affective and Sensory dimensions. This could be because the vagina has natural lubrication, and when it is absent, it can be replaced by artificial lubrication [84]. However, erection problems are more difficult to repair and produce immense frustration as well as an extremely negative psychosocial impact [85,86]. A similar pattern is found in the ease of reaching orgasm and the attitude toward sexual fantasies. In short, whereas in lesbians all psychosexual variables correlate with all ORS dimensions, in men, such a consistent pattern does not exist. These results are in line with previous findings in heterosexual populations, which show that the subjective experience of female orgasm could be more complex and explainable through more variables compared to male orgasm [6,87].

Finally, the ORS has proven to be an optimal tool to discriminate between gay men and women with and without orgasmic difficulties. As hypothesized, people with orgasmic difficulties report lower intensity in the subjective experience of orgasm, as reported by Arcos-Romero et al. [5]. Therefore, the ORS is a scale that–from a clinical viewpoint–can identify the dimensions of the psychological experience of orgasm that are affected in gay people with orgasmic dysfunctions, serving as a treatment guide.

This study has a few limitations. Despite having a large sample, it is not possible to generalize the results to the entire Spanish population, as the participants were selected by incidental sampling and were all cisgender, which excludes gender minorities. Additionally, the battery of questionnaires was disseminated through a social network, which makes participation difficult for people without access to it. Finally, another limitation could be the use of the Kinsey Scale, which only assesses sexual orientation in purely behavioral terms. There are other scales (e.g., the Multidimensional Scale of Sexuality [88]) that might be more appropriate, as they offer a more multifactorial description of sexual orientation. However, despite these limitations, the results obtained are considered to be relevant, both from a research and a clinical perspective. Future research and interventions in the field of sexual health could incorporate the ORS to evaluate the subjective experience of orgasm in the gay population, as well as from an educational viewpoint, where these findings could be incorporated to promote the sexual health of the non-heterosexual collective. To broaden the study of the subjective experience of orgasm, future research should incorporate experiences of trans, intersex, asexual, and LGBTIQA+ people with functional and/or psychic diversity, as well as serodiscordant couples. It is also proposed that future studies incorporate a few personal and interpersonal variables, such as internalized homophobia, whose influence has already been studied in terms of sexual satisfaction in people with same-sex partners [30], partner sexual satisfaction or satisfaction with the relationship, given the association of these latter variables with the subjective experience of orgasm [6].

## 5. Conclusions

In conclusion, the scarcity of research in the non-heterosexual population extends to the fact that there is little evidence about instruments that assess different psychosexual dimensions in people belonging to sexual minorities. On the other hand, there is also a paucity of studies using invariant measures between the two populations. The subjective experience of orgasm is an essential construct within sexual functioning, hence the relevance of validating instruments that assess this dimension also in gays and lesbians. The results obtained in this study indicate that the Spanish version of the ORS is reliable and valid to examine the subjective experience of orgasm in gays and lesbians, as well as to discriminate between gay men and women with and without orgasmic difficulties. This makes it a useful instrument for both research and clinical practice.

## Figures and Tables

**Table 1:** Sociodemographic characteristics of the participants.

	Gay People (*n* = 800)						Heterosexual People (*n* = 800)					
	Men (*n* = 400)		Women (*n* = 400)				Men (*n* = 400)		Women (*n* = 400)			
	Rank	*M* (*SD*)	Rank	*M* (*SD*)	*t*/*χ*^2^	*d*/*V*	Rank	*M* (*SD*)	Rank	*M* (*SD*)	*t*/*χ*^2^	*d*/*V*
Age (years)	18–62	32.07 (9.73)	18–55	29.54 (7.51)	4.76 ***	0.29	18–62	34.41 (12.40)	18–63	32.42 (10.18)	2.48 *	0.18
	***n* (%)**		***n* (%)**				***n* (%)**		***n* (%)**			
Education level												
Primary Education	3 (0.8)		4 (1)		4.21		17 (4.3)		3 (0.8)		23.06 ***	0.17
Secondary Education	52 (13)		53 (13.3)				72 (18.1)		45 (11.3)			
University Degree	304 (76.2)		283 (71.1)				231 (58)		286 (71.7)			
Other	40 (10)		58 (14.6)				78 (19.6)		65 (16.3)			
	***M* (*SD*)**		***M* (*SD*)**				***M* (*SD*)**		***M* (*SD*)**			
Age of first sexual relationship (years)	17.83 (4.13)		17.26 (3.03)		2.25 *	0.16	17.45 (2.91)		16.87 (2.45)		3.01 **	0.22
	***n* (%)**		***n* (%)**				***n* (%)**		***n* (%)**			
Current Relationship												
Yes	224 (56)		316 (79)		48.23 ***	0.25	320 (80)		295 (73.8)		4.40 *	0.07
No	176 (44)		84 (21)				80 (20)		105 (26.3)			
	***M* (*SD*)**		***M* (*SD*)**				***M* (*SD*)**		***M* (*SD*)**			
Duration of relationship with current partner (months)	68.21 (74.75)		51.01 (49.91)		3.00 **	0.28	114.63 (131.81)		85.91 (105.01)		3.00 **	0.24
	***Mₑ***	***M* (*SD*)**	***Mₑ***	***M* (*SD*)**			***Mₑ***	***M* (*SD*)**	***Mₑ***	***M* (*SD*)**		
Number of sexual partners	20	46.57 (64.27)	6	10.24 (14.16)	10.86 ***	0.78	6	11.96 (22.85)	7	13.03 (23.89)	−0.64	
Last orgasm reached with another person												
Through penetrative sex (vaginal/anal/other)	203 (50.8)		45 (11.3)		212.13 ***	0.52	290 (72.5)		195 (48.8)		63.37 ***	0.28
Through oral stimulation of the partner	64 (16)		122 (30.5)				67 (16.8)		76 (19)			
Through manual stimulation of the partner	57 (14.3)		173 (43.3)				22 (5.5)		80 (20)			
Through manual stimulation of yourself with the partner present	68 (17)		26 (6.5)				15 (3.8)		29 (7.3)			
Other	8 (2)		34 (8.5)				6 (1.5)		19 (4.8)			

Note. *M_e_* = median, *M* = mean, *SD* = standard deviation, *t*/*χ*^2^ *=* statistical test, *d*/*V* = effect size. * *p* < 0.05, ** *p* < 0.01, *** *p* < 0.001.

**Table 2:** Factorial invariance according to sex and sexual orientation: gay men, lesbian women, heterosexual men, and heterosexual women.

Model	χ^2^	df	*p*	CFI	TLI	RMSEA	RMSEA 90% CI	ΔCFI
Configural	2620.64	559	<0.001	0.980	0.979	0.047	0.045, 0.048	
Weak	2985.67	1139	<0.001	0.979	0.978	0.046	0.044, 0.048	<0.01
Strong	3227.62	1202	<0.001	0.978	0.978	0.047	0.045, 0.049	<0.01
Strict	3335.46	1277	<0.001	0.977	0.978	0.046	0.044, 0.048	<0.01

Note. CFI = Comparative Fit Index; TLI = Tucker-Lewis Index; RMSEA = Root Mean Square Error of Approximation; CI = Confidence Interval.

**Table 3:** Reliability of the ORS subscales.

Subscales	Gay People		Heterosexual People	
	Men	Women	Men	Women
Affective	0.91	0.92	0.91	0.91
Sensory	0.92	0.94	0.93	0.93
Intimacy	0.88	0.87	0.86	0.82
Rewards	0.85	0.88	0.88	0.86

**Table 4:** Effects of sex on subjective orgasm experience in gay people.

Variables	Men *n* = 386 *M (SD*)	Women *n* = 393 *M (SD*)	*F*_(1, 771)_	*p*	*d*
Affective	25.23 (4.88)	26.68 (4.44)	7.8	0.005	0.31
Sensory	36.14 (13.61)	43.1 (13.97)	44.57	<0.001	2.31
Intimacy	9 (4.3)	11.23 (3.66)	20.6	<0.001	4.28
Rewards	9.53 (3.95)	10.14 (4.04)	0.70	0.403	0.15

Note. *M* = mean, *SD* = standard deviation, *F =* statistical test, *d* = effect size.

**Table 5:** Effects of sexual orientation on subjective orgasm experience.

Variables	Gay Men *n* = 386 *M (SD*)	Heterosexual Men *n* = 392 *M (SD*)	*F*_(1, 770)_	*p*	*d*	Lesbians *n* = 393 *M (SD*)	Heterosexual Women *n* = 391 *M (SD*)	*F*_(1, 776)_	*p*	*d*
Affective	25.23 (4.88)	26.06 (4.23)	0.09	0.767	0.18	26.68 (4.44)	26.6 (3.87)	0.12	0.730	5.77
Sensory	36.14 (13.61)	38.95 (14.05)	1.18	0.279	0.20	43.1 (13.97)	43.04 (13.49)	0.38	0.538	2.82
Intimacy	9 (4.3)	10.47 (3.75)	0.02	0.898	3.89	11.23 (3.66)	9.9 (3.74)	22.95	<0.001	2.77
Rewards	9.53 (3.95)	10.7 (3.87)	11.78	0.001	3.03	10.14 (4.04)	9.99 (4)	0.34	0.559	0.05

Note. *M* = mean, *SD* = standard deviation, *F* = statistical test, *d* = effect size.

**Table 6:** Bivariate correlation matrix for subjective orgasm experience, attitude positive toward sexual fantasy and sexual functioning.

	1	2	3	4	5	6	7	8	9	10
1. Affective	-	0.60 **	0.46 **	0.41 **	0.25 **	0.15 **	0.14 **	0.14 **	0.21 **	0.31 **
2. Sensory	0.61 **	-	0.38 **	0.37 **	0.34 **	0.28 **	0.31 **	0.26 **	0.21 **	0.34 **
3. Intimacy	0.53 **	0.42 **	-	0.53 **	0.15 **	0.12 *	0.15 **	0.12 *	0.15 **	0.19 **
4. Rewards	0.41 **	0.34 **	0.41 **	-	0.15 **	0.18 **	0.17 **	0.12 *	0.12 *	0.20 **
5. Attitude positive toward sexual fantasies	0.10 *	0.19 **	0.08	0.11 *	-	0.32 **	0.36 **	0.20 **	0.15 **	0.13 *
6. Drive	0.15 **	0.24 **	0.11 *	0.08	0.26 **	-	0.75 **	0.51 **	0.42 **	0.40 **
7. Arousal	0.16 **	0.25 **	0.10 *	0.13 **	0.31 **	0.70 **	-	0.55 **	0.47 **	0.43 **
8. Erection/Lubrication	0.13 **	0.15 **	0.09	−0.01	0.15 **	0.29 **	0.40 **	-	0.50 **	0.54 **
9. Orgasm	0.11 *	0.10	0.09	0.13 *	0.20 **	0.26 **	0.31 **	0.46 **	-	0.69 **
10. Satisfaction from orgasm	0.34 **	0.20 **	0.22 **	0.20 **	0.27 **	0.39 **	0.36 **	0.34 **	0.61 **	-

Note. Values below the diagonal are based on gay men scores. Values above the diagonal are based on lesbians scores. ** *p* < 0.01. * *p* < 0.05.

## Data Availability

The data presented in this study are available on request from the corresponding author. The data are not publicly available due to privacy.
